# Cytokine Production by Leukocytes of Military Personnel with Depressive Symptoms after Deployment to a Combat-Zone: A Prospective, Longitudinal Study

**DOI:** 10.1371/journal.pone.0029142

**Published:** 2011-12-14

**Authors:** Mirjam van Zuiden, Cobi J. Heijnen, Rens van de Schoot, Karima Amarouchi, Mirjam Maas, Eric Vermetten, Elbert Geuze, Annemieke Kavelaars

**Affiliations:** 1 Laboratory of Neuroimmunology and Developmental Origins of Disease (NIDOD), University Medical Center, Utrecht, Utrecht, the Netherlands; 2 Research Centre – Military Mental Health, Ministry of Defence, Utrecht, the Netherlands; 3 Department of Methodology and Statistics, Faculty of Social Sciences, Utrecht University, Utrecht, the Netherlands; 4 Department of Psychiatry, Rudolf Magnus Institute of Neuroscience, University Medical Center Utrecht, Utrecht, the Netherlands; Emory University School of Medicine, United States of America

## Abstract

Major depressive disorder (MDD) is frequently diagnosed in military personnel returning from deployment. Literature suggests that MDD is associated with a pro-inflammatory state. To the best of our knowledge, no prospective, longitudinal studies on the association between development of depressive symptomatology and cytokine production by peripheral blood leukocytes have been published. The aim of this study was to investigate whether the presence of depressive symptomatology six months after military deployment is associated with the capacity to produce cytokines, as assessed before and after deployment. 1023 military personnel were included before deployment. Depressive symptoms and LPS- and T-cell mitogen-induced production of 16 cytokines and chemokines in whole blood cultures were measured before (T0), 1 (T1), and 6 (T2) months after return from deployment. Exploratory structural equation modeling (ESEM) was used for data reduction into cytokine patterns. Multiple group latent growth modeling was used to investigate differences in the longitudinal course of cytokine production between individuals with (n = 68) and without (n = 665) depressive symptoms at T2. Individuals with depressive symptoms after deployment showed higher T-cell cytokine production before deployment. Moreover, pre-deployment T-cell cytokine production significantly predicted the presence of depressive symptomatology 6 months after return. There was an increase in T-cell cytokine production over time, but this increase was significantly smaller in individuals developing depressive symptoms. T-cell chemokine and LPS-induced innate cytokine production decreased over time and were not associated with depressive symptoms. These results indicate that increased T-cell mitogen-induced cytokine production before deployment may be a vulnerability factor for development of depressive symptomatology in response to deployment to a combat-zone. In addition, deployment to a combat-zone affects the capacity of T-cells and monocytes to produce cytokines and chemokines until at least 6 months after return.

## Introduction

Mental health disorders frequently diagnosed in military personnel after deployment include major depressive disorder (MDD). Prevalence estimates for MDD range from 7.3% to 15.9% in US infantry soldiers 12 months after return from deployment to Iraq [1]. A number of studies have investigated the capacity of peripheral leukocytes of individuals with MDD or depressive symptoms to produce cytokines after in vitro stimulation. Increased [2]–[7], unaltered [8]–[12] and decreased [2], [13], [14] mitogen-induced pro-inflammatory cytokine production by leukocytes from individuals with MDD or depressive symptoms compared to non-depressed controls have been reported. However, in the majority of these studies only a small number of predominantly innate pro-inflammatory cytokines has been investigated. In addition, all of these studies have been performed within a cross-sectional design, with a different ‘time since onset’ of the depressive symptoms. Therefore, it is as yet unknown whether a causal relation exists between the development of MDD and the capacity to produce cytokines.

The potential involvement of inflammatory mediators in depression is underscored by the observation that individuals with lifetime MDD have epigenetic changes in methylation of inflammation-associated genes [15]. In addition, meta-analyses investigating the association between MDD and circulating levels of C-reactive protein (CRP), innate pro-inflammatory cytokines IL-6, TNF-α, IL-1, and the IL-1 receptor antagonist showed that these inflammatory markers are increased in MDD [16], [17]. These effects were present within both clinical and community samples, and in studies using clinical interviews and studies using self-report measures [16]. Furthermore, higher amounts of circulating IL-2 soluble receptors (s-IL2-r) [18]–[20] have been observed in individuals with MDD or depressive symptomatology.

In the current study our aim was to determine whether the level of mitogen-induced cytokine production before and/or after deployment was associated with the presence of a high level of depressive symptoms 6 months after return from military deployment. We used a prospective, longitudinal design, in which data were collected before, as well as 1 and 6 months after deployment to a combat-zone. We investigated the production of a broad range of innate and T-cell cytokines, including pro- and anti-inflammatory cytokines, as well as chemokines. There is functional overlap between cytokines, and chance capitalization for type-I errors will occur when testing 16 longitudinal models. Therefore, we performed data reduction by using exploratory structural equation modeling (ESEM), which is a recently developed statistical method in which exploratory factor analysis is performed within a structural equation modeling setting [21]. Subsequently, differences in the longitudinal course of cytokine production between individuals with and without depressive symptomatology 6 months after deployment were investigated using multiple group latent growth modeling (LGM) [22]. In addition, logistic regression analysis was performed to test the predictive value of cytokine production for the presence of depressive symptomatology after deployment.

## Materials and Methods

### Ethics Statement

The study was approved by the Institutional Review Board of the University Medical Center Utrecht, the Netherlands. Written informed consent was obtained after participants got a written and verbal description of the study.

### Participants

Military personnel of the Dutch Armed Forces assigned to a 4-month deployment to Afghanistan were included in this study. Duties during deployment included combat patrols, clearing or searching homes and buildings, participation in de-mining operations, and transportation across enemy territory. They were exposed to typical war-zone stressors such as exposure to enemy fire, armed combat, and seeing seriously injured and dead fellow soldiers and civilians (including women and children).

Participants were assessed 1 to 2 months prior to deployment (T0) and approximately 1 (T1) and 6 months (T2) after their return. At each assessment, participants filled out several ‘paper-and-pencil’ questionnaires. In addition, a heparinized blood sample was drawn between 8.00 and 11.30 a.m. Heparinized blood was kept at room temperature. Data were collected between April 2005 and September 2009.

We included 1023 participants before deployment. Twenty-eight participants (2.5%) were not available for follow-up (non-deployed (n = 26); deceased during deployment (n = 2)). Of the eligible 995 participants, 825 completed the assessment at T1 (82.9%) and 749 completed the assessment at T2 (75.3%). Compared to eligible individuals who completed the T2 assessment, dropouts were younger during deployment (mean (SD): dropouts: 26.03 (7.08); completers: 29.09 (9.24), t_(976)_: −4.604, p<.001). Consequently they had been deployed less often (mean (SD): dropouts: 0.55 (0.88); completers: 0.93 (1.23), t_(886)_: −4.03, p<.001) and were lower ranked (χ^2^_(3)_: 21.656, p<.001). There were no significant differences in gender distribution (χ^2^_(1)_: 1.738, p = .187) and educational level (χ^2^_(2)_: 5.922,p = .052). In addition, there were no differences in pre-deployment questionnaire scores for depression (t_(821)_: 0.385, p = .701) and PTSD (t_(682)_: 0.247, p = .805).

Depression scores at T2 were missing for 16 participants (2.1%). The remaining 733 participants were divided into two groups based on their level of depressive symptoms at T2. Participants were assigned to the depressive symptoms group when their score on the Symptom Checklist (SCL-90) depression subscale was ≥24 at T2 (n = 68) [23]. This cut-off corresponds to the mean plus 2 standard deviations (95^th^ percentile) on the SCL-90 depression subscale within a population of 840 Dutch military personnel (mean (SD): 18.06 (3.15)). Participants scoring below the cut-off on the SCL-90 depression subscale at T2 were assigned to the non-depressed group (n = 665).

### Questionnaires

Level of depressive symptoms over the past week was assessed with the Dutch version of the Symptom Checklist (SCL-90) depression subscale [24]. This subscale consists of 16 items ranging from 1 (not at all) to 5 (very much). The total depressive symptom score is the sum score for all items (range 16–80). A higher score indicates more depressive symptoms. The questionnaire has good reliability and is frequently used within research and clinical settings. The validity of the subscale as a screening instrument for MDD has been shown in primary care patients [25], and in the aftermath of stroke [26] and myocardial infarction [27].

Depression and PTSD are frequently co-morbid [28]. Therefore, the level of posttraumatic stress disorder (PTSD) symptoms over the past 4 weeks was assessed with the Dutch 22-item Self-Report Inventory for PTSD (SRIP). The questionnaire consists of three subscales representing the PTSD symptom clusters re-experiencing, avoidance and hyper-arousal. The total PTSD score is the sum score for all items (range: 22–88). The SRIP is well validated and has good concurrent validity with other PTSD measures such as the Clinician Administered PTSD Scale (CAPS) and Mississippi scale for PTSD [29], [30]. Exposure to deployment-stressors was assessed with a 13-item checklist [31].

Collected demographics included age during deployment, sex, body height, weight, smoking, alcohol use, and use of possibly interfering medication (non-systemic glucocorticoids (nasal spray or crème), antihistamines, cholesterol lowering medications and antihypertensive medication). Body Mass Index (BMI) was calculated by dividing body weight by the square of body height (kg/m^2^).

### Cytokine production

#### CD2/CD28-induced T cell cytokine production

Whole blood, diluted 1∶10 with RPMI-1640 (Gibco, Grand Island, NY), 100 U/ml penicillin, 100 µg/ml streptomycin and 2 µM L-glutamine was stimulated with the T-cell mitogen anti-CD2/CD28 monoclonal antibodies (CLB, Amsterdam, Netherlands, final concentration anti-CD2.1/anti-CD2.2 0.33 µg/ml and anti-CD28 1.33 µlg/ml) for 72 hours at 37° C/5% CO2 in 96-well round-bottomed plates [32], [33]. T cell mitogen-induced secretion of interleukin (IL)-2, IL-4, IL-5, IL-6, IL-10, TNF-α, monocyte chemoattractant protein (MCP)-1 (CCL2), interferon-gamma induced protein (IP)-10 and RANTES (CCL5) were measured in supernatants using multiplex cytokine assay as described before [34]–[37]. IFN-γ was analyzed by ELISA (CLB, Amsterdam, the Netherlands).

#### Lipopolysaccharide-induced innate cytokine production

Whole blood, diluted 1∶10 with RPMI-1640 (Gibco, Grand Island, NY), was stimulated with Lipopolysaccharide (LPS, Escherichia Coli 0127:B8, Sigma, final concentration 1 ng/ml) for 24 hours at 37° C/5% CO2 in 96-well flat-bottomed plates to activate cytokine production. Supernatants were analyzed by using multiplex assay for the presence of IL-1α, IL-1β, IL-6, IL-8, IL-10 and TNF-α, as described previously [37].

#### FACS analysis

Leukocyte subsets in peripheral blood were assessed using dual colour fluorescence analysis with a Becton Dickinson Calibur flowcytometer. Whole blood was stained using monoclonal antibodies labelled with either fluorescine isothiocyanate or phyco-erythrin to quantify CD14+ (monocytes), CD3+ (total T-cells), CD4+ (T-helper/inducer) and CD8+ (T-cytotoxic/suppressor-effector) cells. Absolute numbers of cells were calculated from a total leukocyte count.

### Data analysis

Basic statistical analyses were conducted using SPSS 15.0. Exploratory structural equation modeling (ESEM), multiple group latent growth modeling and logistic regression analysis were performed using Mplus 6.1 [38]. Immune parameters and questionnaire scores were tested for normality and transformed when necessary (see Table 1 for applied transformations). A limited number of missing values in the immune parameters were present due to technical and handling problems (cytokines: T0: 0.91%, T1: 1.46%, T2: 2.00%; cell subsets: T0: 1.68%, T1: 1.41%, T2: 2.93%). Outliers in the immune parameters were removed if z-values were outside the range of ±3.29 [39] (cytokines: T0: 0.57%, T1: 0.80%, T2: 0.89%; cell subsets: T0: 1.17%, T1: 0.85%, T2: 0.45%).

**Table 1:** CD2/CD28- and LPS- induced cytokine and chemokine production before (T0), 1 (T1) and 6 (T2) months after deployment.

	T0			T1			T2		
	Mean	SD	N	Mean	SD	N	Mean	SD	N
**CD2/CD28**									
IL-2 a	1082.6	1369.1	995	1243.9	1591.0	819	1387.1	1654.1	743
IL-4 a	79.2	84.5	1002	115.2	129.4	814	125.2	135.0	742
IL-5 a	613.1	1008.1	983	642.4	1026.0	811	715.2	1088.4	736
IL-6 a	1229.5	1348.6	991	977.1	831.0	810	937.1	843.6	742
IL-10 ^b^	432.7	356.8	999	543.3	454.4	817	589.1	440.7	744
TNF-α a	560.8	475.7	1000	837.0	853.1	802	1209.3	1089.8	733
IFN-γ a	20593.2	23593.4	998	21117.9	21951.8	764	28376.0	34652.1	708
MCP-1 a	18308.0	14514.0	998	14577.4	10102.1	817	12728.2	8818.9	741
IP-10 ^b^	5977.5	3485.5	994	7355.7	4539.4	818	9099.8	5977.1	744
RANTES a	7142.0	6893.1	995	6298.5	4833.4	815	5069.5	4514.0	741
**LPS**									
IL1-α a	68.3	51.1	1002	60.1	47.0	793	57.0	41.4	721
IL1-β ^b^	314.4	227.4	996	260.6	179.0	802	241.1	156.6	730
IL-6 ^b^	2951.7	1812.7	1008	2492.8	1988.3	806	2201.1	1286.4	712
IL-8 ^b^	1955.9	1144.3	1006	2048.0	1087.3	805	2069.7	1076.8	725
IL-10 a	33.8	35.6	1006	28.8	25.5	805	31.0	28.7	733
TNF-α a	851.5	677.9	1005	578.7	467.5	796	489.2	385.7	729

a log10-transformation applied; ^b^ square root-transformation applied.

#### Exploratory structural equation modelling

For data reduction of the cytokines, exploratory structural equation modeling (ESEM) was used [21]. Within this recently developed statistical method, exploratory factor analysis (EFA) is performed within a structural equation modeling (SEM) setting. Combining EFA with SEM provides the opportunity to assess goodness-of-fit indices and measurement invariance of the factor solution across time or groups, which was previously not possibly within EFA. ESEM is more appropriate to model complex biological data than conventional confirmatory factor analysis (CFA): whereas CFA only provides adequate model fit with simple structure (i.e. each indicator loads on one pre-determined factor), in ESEM all indicator loadings on all factors are estimated by default [21].

ESEM was performed, using data from T0 of all participants with 1–5 specified factors, using maximum likelihood estimation. To select the best fitting model the Aikake information criterion (AIC [40]) and Bayesian information criterion (BIC [41]) of the models were compared. The model with the smallest AIC and BIC was chosen. Additionally, the comparative fit index (CFI), Tucker-Lewis index (TLI), root mean square error of approximation (RMSEA) and standardized root mean square residual (SRMR) were used to test the goodness-of-fit of the models. Adequate fit was defined as CFI>0.9, TLI>0.9, RMSEA<0.08, and SRMR<0.08 [42].

Subsequently, it was investigated whether the chosen ESEM model for T0 was stable (i.e. measurement invariant) across time. For reliable estimation of differences in mean factor scores across time, the presence of scalar and metric measurement invariance (i.e. similar factor loadings and item means across time) is a prerequisite [42], [43]. For this purpose ESEM with target rotation was performed, using data from T0–T2. Insignificant factor loadings at T0 were estimated at 0 in the target model to simplify the final model [21].

To deal with missing data, full information maximum likelihood estimation (FIML) was used, which includes all available data in the model. Thus, individuals with missing time points or missing values within time points were retained in the analyses. This provides more reliable estimates compared to other methods of handling missing data, such as list-wise deletion or mean imputation [44].

#### Multiple group latent growth modelling

Within latent growth modeling, the average starting point (intercept) and average change over time (slope) are estimated in longitudinal data [22]. In multiple group latent growth modeling (LGM), models are estimated simultaneously across groups. Using multiple group LGM we investigated whether the intercept and slope of questionnaire scores, cytokine production (factor scores) and cell subsets differed between participants with and without a high level of depressive symptoms at T2. A two-step model was used. First, means of the intercepts and slopes were freely estimated across groups. Then, a model was examined in which the intercept and slope were constrained to be equal for both groups. Chi-square difference testing (Δ χ^2^) was used to formally test whether the more constrained model fitted the data as well as the non-constrained model: a significant Δ χ^2^ indicates that the more constrained model fits the data significantly worse [38]. When significant group differences in intercept and slope were found, the association between possibly confounding variables and the intercept and/or slope of both groups was tested.

#### Logistic regression analysis

Logistic regression analysis was performed to test the predictive value of cytokine factor scores for the presence of depressive symptomatology. In the regression analysis, pre-deployment SCL-90 depression score and BMI, which differed between groups, were included to determine that the observed association between cytokine factors scores and depression were not confounded by these variables. To be able to compare the odds ratios associated with the included variables, variables were standardized (mean (SD): 0(1)).

## Results

### Participant characteristics

Participants were assessed 1 to 2 months before a 4-month military deployment to Afghanistan (T0), and 1 (T1) and 6 (T2) months after return from deployment. 68 (9.3%) participants reported a high level of depressive symptoms at T2. The intensity and frequency of depressive symptoms increased over time in the group with depressive symptoms at T2, while the mean depressive symptom score of the non-depressed group decreased slightly over time. The group with a high level of depressive symptoms at T2 already had more depressive symptoms before deployment than the non-depressed group (depressive symptoms group: intercept: 1.327(0.013), p<.001; slope: 0.010(0.001), p<.001. non-depressed group: intercept: 1.241 (0.002), p<.001; slope: −0.001 (0.000), p = .004; Δ χ^2^_(3)_: 130.939, p<.001).

Individuals with depressive symptoms at T2 reported higher levels of PTSD symptoms at T2 (t_(726)_: −17.546, p<.001), although the variance in SRIP total scores was considerable. In addition, individuals with a high level of depressive symptoms at T2 reported a larger number of deployment stressors, including a larger number of combat experiences (Table 2). Furthermore, BMI at T0 was higher in individuals who had depressive symptoms at T2 (t_(675)_: −2.203, p = .028).

**Table 2:** Questionnaire scores and deployment, demographic and pre-deployment characteristics of the total sample, and the sample divided based on depressive symptoms 6 months after return from deployment.

	Total sample(n = 1023)	Depressive symptoms6 months after deployment (n = 68)	No depressivesymptoms6 months afterdeployment (n = 665)	P
SCL-90 depression score T0(range: 16–80)	18.06 (3.15)	22.15 (5.47)	17.55 (2.33)	<.001
SCL-90 depression score T1(range: 16–80)	18.30 (3.83)	24.63 (5.87)	17.71 (3.10)	<.001
SCL-90 depression score T2(range: 16–80)	18.34 (4.16)	29.12 (5.39)	17.24 (1.75)	<.001
SRIP (PTSD) total score T2(range: 22–88)	27.79 (7.15)	40.44 (10.88)	26.54 (5.21)	<.001
Nr. Of deployment stressors (range: 0–13)	4.77 (2.55)	5.63 (2.40)	4.75 (2.55)	.013
Age during deployment	28.44 (8.89)	29.54 (8.84)	28.98 (9.20)	.629
Nr. Of previous deployments	0.83 (1.16)	1.08 (1.31)	0.92 (1.23)	.268
Gender				.729
Male	931 (91.0%)	61 (98.7%)	605 (91%)	
Female	92 (9.0%)	7 (10.3%)	60 (9%)	
Education				.675
Low	371 (40.0%)	29 (44.6%)	242 (39.2%)	
Middle	454 (48.9%)	28 (43.1%)	299 (48.5%)	
High	103 (11.1%)	8 (12.3%)	76 (12.3%)	
Rank during deployment				.844
Soldier	400 (40.2%)	24 (35.3%)	246 (37.1%)	
Corporal	206 (20.7%)	16 (23.5%)	134 (20.2%)	
Non-commissioned officer	255 (25.7%)	20 (29.4%)	185 (27.9%)	
Officer	133 (13.4%)	8 (11.8%)	98 (14.8%)	
BMI T0	24.71 (2.78)	25.54 (3.41)	24.70 (2.74)	.028
Smoking T0 (yes)	407 (44.5%)	28 (43.8%)	258 (42.2%)	.814
Alcohol use T0				.786
None	93 (10.3%)	7 (11.3%)	63 (10.5%)	
0–20 units/week	754 (83.9%)	50 (80.6%)	502 (83.5%)	
>20 units/week	52 (5.8%)	5 (8.1%)	36 (6.0%)	
Medication use T0 (yes)	56 (5.5%)	3 (4.4%)	42 (6.3%)	.533
Season of assessment T0				.181
Spring	343 (33.5%)	27 (39.7%)	204 (30.7%)	
Summer	102 (10.0%)	9 (13.2%)	67 (10.1%)	
Autumn	308 (30.1%)	13 (19.1%)	202 (30.4%)	
Winter	270 (26.4%)	19 (27.9%)	192 (28.9%)	

### Data reduction of cytokine parameters

We determined 10 CD2/CD28-induced and 6 LPS-induced cytokines before (T0), 1 (T1) and 6 months (T2) after deployment. Non-transformed values and applied transformations for the CD2/CD28- and LPS-induced cytokine production are reported in Table 1. We performed data reduction by exploratory structural equation modeling (ESEM) on the data from T0 of all participants. Correlation matrices of the immune parameters revealed that IL-8 was only weakly correlated with the other variables (strongest correlation -.13 with MCP1). IL-8 was therefore not included in the factor analyses. We analyzed consecutive ESEM models with 1–5 factors respectively. According to the AIC and BIC measures, the 5-factor model provided the best fit (Table S1). However, within this model the estimated residual variance for CD2/CD28-induced IL-4 was negative, and therefore this variable was removed from the model [45]. We reran the 5-factor model without CD2/CD28-induced IL-4 and obtained a better fit compared to the 5-factor model including IL-4 (AIC = 34271, BIC = 34704). Within this model the estimated factor loading for LPS-induced IL-6 was larger than 1. This cytokine only loaded on one factor, with small factor loadings from the other variables on this factor (largest standardized loading:.044), and was removed from the model as suggested [45]. We then analyzed a 4 factor model without CD2/CD28-induced IL-4 and LPS-induced IL-6. The model had good fit and lower AIC/BIC than the previous model (CFI = .962, TLI = .908, RMSEA = .086, SRMR = .026, AIC = 27052, BIC = 27406). The factor structure was similar at T1 and T2 compared to T0 (i.e. model with scalar and metric measurement invariance fitted the data better than the free model) (model fit: CFI = .862, TLI = .835, RMSEA = .072, SRMR = .074, AIC = 63043, BIC = 64015).

The first factor that emerged out of the ESEM including the data from T0–T2 contained CD2/CD28-induced cytokines. Given the evidence that CD2/CD28 is a strong activator of T-cells [33], we refer to Factor 1 as T-cell cytokine production. Since CD2 is also expressed on NK-cells, NK-cells may also contribute to the production of the cytokines in this factor. The second factor included all CD2/CD28-induced chemokines and CD2/CD28 induced IL-6 (referred to as T-cell-induced chemokine/IL-6 production). The third factor included all LPS-induced cytokines (referred to as innate cytokine production). The fourth factor contained CD2/CD28-induced IP-10 with lower loadings of CD2/CD28-induced TNF-α and RANTES and LPS-induced IL-10. This factor could not be interpreted in a functional way, and therefore we chose not to include it in the subsequent analyses. The final factor solution is depicted in Table 3. Most cytokines had significant cross-loadings, underscoring the appropriateness of using an ESEM model.

**Table 3:** Final exploratory structural equation _odelling (ESEM) factor solution.

	*Factor 1*	*Factor 2*	*Factor 3*	*Factor 4*
	T-cellcytokines	T-cell-induced chemokines/IL-6	Innatecytokines	Residualfactor
**CD2/CD28**				
IL-2	**.815**	−.048	.013	−.071
IL-5	**.663**	.044	−.036	.010
IL-6	**.462**	**.488**	.024	−.107
IL-10	**.662**	−.154	.000	.094
TNF-α	**.812**	.142	.054	.206
IFN-γ	**.867**	.001	.022	−.068
MCP-1	−.019	**.835**	.137	−.014
IP-10	**.491**	**.388**	.000	**.447**
RANTES	**.455**	**.518**	−.063	−.258
**LPS**				
IL-1α	−.061	**−.322**	**.837**	.173
IL-1β	.001	.001	**.700**	.001
IL-10	−.068	−.137	**.507**	.232
TNF-α	−.060	.054	**.715**	−.180

Indicator loadings >.3 are depicted in bold.

### Longitudinal trajectory of mitogen-induced T-cell cytokine production

We investigated whether the longitudinal course of T-cell cytokine production (Factor 1) differed between the group with high levels of depressive symptoms at T2 and the non-depressed group. The multiple group latent growth model in which the intercept and slope of both groups were constrained to be equal, fitted the data less well (AIC = 4975, BIC = 5039) (Δ χ^2^_(2)_ = 8.784, p = .012) than the model in which the intercept and slope were freely estimated for both groups (CFI = .990, TLI = .971, RMSEA = .101, SRMR = .020, AIC = 4970, BIC = 5043). Additional models constraining the intercept and slope separately confirmed that both intercept and slope differed between the two groups (intercept-model: Δ χ^2^_(1)_: 7.449, p = .006; slope-model: Δ χ^2^_(1)_: 4.395, p = .036).

**Figure 1:**
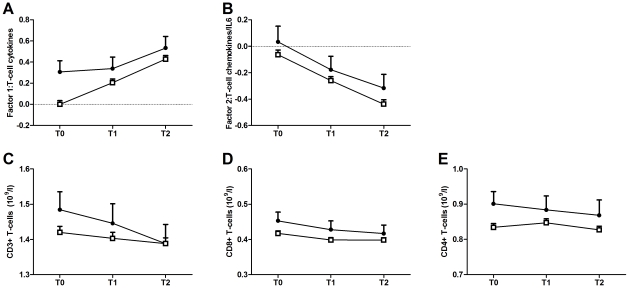
Longitudinal course of T-cell cytokine production and T-cell counts. Longitudinal course of CD2/CD28-induced Factor 1: T-cell cytokine production (A); CD2/CD28-induced Factor 2: T-cell chemokine/IL-6 production (B) and CD3+ T-cell counts (C); CD8+ cytotoxic/suppressor-effector T-cell counts (D) and CD4+ T helper-cell counts (E), as assessed before deployment (T0), 1 month (T1) and 6 months (T2) after return from deployment. The course of the group with depressive symptoms at T2 (n = 69) is depicted by the black circles (mean + SEM). The course of the group without depressive symptoms at T2 (n = 664) is depicted by the white rectangles (mean + SEM).

Before deployment, the group with depressive symptoms at T2 already had higher T-cell cytokine production than the non-depressed group (depressive symptoms group: 0.296 (0.103), p = .004; non-depressed group: −0.016 (0.036), p = .661). T-cell cytokine production increased over time for both groups, but the magnitude of the increase was significantly smaller in the depressed group (depressive symptoms group: 0.018 (0.007), p = .003; non-depressed group: 0.033 (0.003), p<.001) (Figure 1A).

Since the group with depressive symptoms at T2 also had a higher level of PTSD symptoms at T2, we investigated whether this might have influenced the observed association between depressive symptoms and T-cell cytokine production. PTSD symptoms at T2 were not significantly associated with the intercept and slope of T-cell cytokine production in both groups (depressive symptoms group: intercept: 0.383 (0.871), p = .660; slope: 0.027 (0.053), p = .610; non-depressed group: intercept: −0.469 (0.483), p = .0332; slope: 0.036(0.036), p = .316).

In addition, since the group with depressive symptoms reported a higher number of deployment stressors, we investigated whether the group difference in experienced deployment stressors might explain the difference in change in cytokine production over time between the groups. However, the number of reported deployment-stressors was not significantly associated with the slope of T-cell cytokine production in both groups (depressive symptoms group: −0.003 (0.003), p = .254; non-depressed group: −0.001 (0.001), p = .257). These findings indicate that the group difference in the trajectory of cytokine production over time cannot be attributed to differences in the number of experienced deployment stressors.

We investigated whether the observed higher level of T-cell cytokine production in the depressed group was associated with group differences in the longitudinal course of total numbers of T-cells (CD3+), cytotoxic/suppressor-effector T-cells (CD8+), and T-helper cells (CD4+). The intercept and slope of the cell subsets did not differ between groups for CD3+ T-cells (Δ χ^2^_(2)_ = 2.038, p = .361), CD8+ cytotoxic/suppressor-effector T-cells (Δ χ^2^_(2)_ = 4.189, p = .123), and CD4+ T-helper cells (Δ χ^2^_(2)_ = 2.088, p = .352) (Figure 1CDE). In addition, the increased T-cell cytokine production over time was not caused by an increase in the number of T-cells or T-cell subsets over time. The number of CD8+ cytotoxic/suppressor-effector T-cells decreased over time within both groups (depressive symptoms group: −0.001 (0.000), p = .018; non depressed group: 0.000 (0.000), p = .002 (standardized estimate: −0.162). The total number of T-cells (depressive symptoms group: −0.007 (0.004), p = .115; non-depressed group: −0.002 (0.001), p = .132) and T-helper cells did not significantly change over time (depressive symptoms group: −0.001 (0.003), p = .819; non-depressed group: 0.000 (0.000), p = .899).

### Longitudinal trajectory of mitogen-induced T-cell chemokine/IL6 production

Next, we investigated whether the longitudinal course of T-cell-induced chemokine/IL-6 production (Factor 2) differed between the group with high levels of depressive symptoms at T2 and the non-depressed group. T-cell chemokine production decreased over time for both groups (depressive symptoms group: −0.027(0.008), p = .001; non-depressed group: −0.028 (0.003), p<.001) (Figure 1B). Comparison of the models with freely estimated (AIC = 4875, BIC = 4948) and constrained intercepts and slopes (CFI = 1.000, TLI = .1.000, RMSEA = .000, SRMR = .022, AIC = 4872, BIC = 4936) showed that the intercept and slope did not significantly differ between the two groups (Δ χ^2^_(2)_ = 1.258, p = .533).

### Longitudinal trajectory of mitogen-induced innate cytokine production

**Figure 2:**
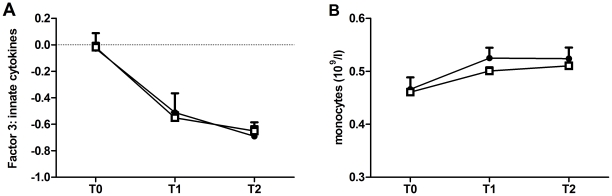
Longitudinal course of innate cytokine production and monocytes. Longitudinal course LPS-induced Factor 3: innate cytokine production (A); and monocyte counts (B), as assessed before deployment (T0), 1 month (T1) and 6 months (T2) after return from deployment. The course of the group with depressive symptoms at T2 (n = 69) is depicted by the black circles (mean + SEM). The course of the group without depressive symptoms at T2 (n = 664) is depicted by the white rectangles (mean + SEM).

Innate cytokine production (Factor 3) decreased over time for both groups (depressive symptoms group: −0.052 (0.010), p<.001; non-depressed group: −0.051 (0.003), p<.001) (Figure 2A). The model fit of the models with freely estimated intercepts and slopes (AIC = 5726, BIC = 5749) was not significantly better than the model fit of the model with constrained intercepts and slopes (CFI = 0.974, TLI = .0.961, RMSEA = .090, SRMR = .037, AIC = 5722, BIC = 5787) (Δ χ^2^_(2)_ = 0.592, p = .745). Therefore we concluded that there were no differences in intercept and slope between groups.

The decrease in innate cytokine production over time was not due to a decrease in total numbers of monocytes over time. In fact, the total number of monocytes significantly increased over time within both groups (depressive symptoms group: 0.002 (0.000), p<.001; non-depressed group: 0.001 (0.000), p<.001) (Figure 2B). There were no differences in the intercept and slope between depressed and non-depressed individuals (fit unconstrained model: AIC = 6311, BIC = 6240; fit constrained model: CFI = .992, TLI = .988, RMSEA = .048, SRMR = .026, AIC = 6312, BIC = 6250; Δ χ^2^_(2)_ = 2.980, p = .225).

### Predictive value of T-cell cytokine production at T0 for depressive symptomatology at T2

Multiple group LGM revealed that individuals with high levels of depressive symptoms had higher T-cell cytokine production at T0, compared to individuals who did not have depressive symptoms at T2. The predictive value of T-cell cytokine production at T0 for the presence of a high level of depressive symptoms at T2 was investigated using logistic regression analysis. We also included depressive symptoms and BMI at T0, to ascertain that these pre-deployment group differences did not confound our results. T-cell cytokine production at T0 significantly and independently predicted the presence of depressive symptomatology at T2 (estimate (SE): 0.615 (0.191), p = .001): with each standard deviation increase in T-cell cytokine production at T0 the odds for the presence of a high level of depressive symptoms at T2 increased approximately 1.9-fold (Odds Ratio: 1.850). In addition, depressive symptoms at T0 also significantly predicted the presence of a high level of depressive symptoms at T2 (Odds ratio for 1 SD increase: 2.955; estimate (SE): 1.083 (0.141), p<.001). Pre-deployment BMI was not significantly associated with the presence of depressive symptoms at T2 (Odds ratio for 1 SD increase: 1.254; estimate (SE): 0.226 (0.141), p = .103).

## Discussion

This is the first prospective, longitudinal study in which associations between the capacity of T-cells and monocytes to produce cytokines and the development of depressive symptoms in response to a period of severe stress have been investigated. We aimed to investigate whether T-cell and monocyte cytokine production represent vulnerability factors for the development of a high level of depressive symptoms in response to a period of severe stress.

To the best of our knowledge, the association between depression and the capacity of monocytes and T-cells to produce cytokines has only been investigated using cross-sectional designs. Moreover, these cross-sectional studies predominantly determined innate cytokines and investigated only a limited range of pro-inflammatory cytokines. We used a unique design: within a large cohort of participants, three assessments were performed, spanning a time period from approximately 1 month before deployment to a combat-zone, until approximately 6 months after return. Moreover, we investigated the production of a broad range of LPS- and CD2/CD28-induced pro- and anti-inflammatory cytokines, and also included several chemokines in our analyses.

The majority of our participants appeared to be resilient and reported no depressive symptoms six months after deployment. However, 10% of the participants reported a high level of depressive symptoms six months after deployment. We aimed to investigate whether these participants with high levels of depressive symptoms had different longitudinal trajectories in cytokine production than the participants who did not become depressed. For this purpose, we decided to use a dichotomous approach in which participants were divided into groups with high and low levels of depressive symptoms six months after deployment (i.e. a score above or below cut-off on the SCL90 depression subscale).

Our results show that individuals with a high level of depressive symptoms 6 months after return from deployment already had higher T-cell mitogen-induced cytokine production before deployment compared to individuals without depressive symptoms 6 months after return. Moreover, T-cell cytokine production before deployment was a significant predictor of the presence of depressive symptoms after deployment. Although the group with depressive symptoms after deployment already had a larger mean depression score before deployment, the observed predictive value of the T-cell cytokines was independent of the severity of depressive symptoms before deployment. Thus, our findings indicate that high mitogen-induced T-cell cytokine production before deployment is a pre-existing vulnerability factor for the development of depressive symptoms in response to a period of severe stress, in this case military deployment. In addition, the observed high T-cell cytokine production was not caused by the presence of posttraumatic stress symptoms in a subset of participants with depressive symptoms: the depressive group did report a higher level of posttraumatic stress symptoms 6 months after deployment, but the T-cell cytokine production was unrelated to the severity of these posttraumatic stress symptoms.

We did not observe differences in the number of T-cells (CD3+, CD4+ and CD8+) before deployment between individuals with and without depressive symptoms after deployment. Hence, the observed higher T-cell cytokine production before deployment in individuals with depressive symptoms after deployment is not caused by higher numbers of T-cells and T-cell subsets in the blood samples obtained from these individuals. Therefore, we propose that the higher T-cell cytokine production in individuals with depressive symptoms reflects a higher capacity of T-cells to produce cytokines that may be related to an increased activation status of these cells.

Higher circulating serum levels of soluble receptors for the T-cell cytokine IL-2 (sIL-2r) have been observed previously in individuals with MDD and depressive symptomatology compared to non-depressed individuals [18]–[20]. In addition, in individuals with cancer, treatment with high doses of the T cell cytokine IL-2 induces a cluster of mood and cognitive symptoms, overlapping with MDD [46] and the depressive symptoms observed in our study, in up to 60% of treated individuals, depending on the dose and modality of treatment [47]–[49]. Interestingly, higher levels of circulating T-cell cytokines in serum were found to be predictive for the development of MDD in response to cytokine administration: higher levels of circulating sIL-2r, and of the anti-inflammatory cytokine IL-10 [50] before the start of IFN-α treatment were associated with increased risk for development of MDD in response to the treatment. These previous findings support our observation of the involvement of both pro- and anti-inflammatory T-cell cytokines in the development of depressive symptomatology.

Our data may also add to the theory that increased cytokine production leads to increased signalling to brain structures involved in the development of depressive symptomatology [48]. The pro-inflammatory cytokine IFN-γ had the highest loading on our T-cell cytokine factor, and therefore had the largest contribution to the score of each participant on the T-cell cytokine factor. Studies on inflammation-induced depressive-like behavior in mice have shown that IFN-γ may be a pivotal mediator in the development of depressive-like behavior, since the development of depressive-like behavior after immune activation by Bacille Calmette-Guerin (BCG) was completely attenuated in IFN-γ receptor knock-out mice [51]. Indoleamine 2,3-dixoygenase (IDO) is a likely intermediate in this relationship [for review see 47, 48, 52]. This tryptophan metabolising enzyme is upregulated by pro-inflammatory cytokines like IFN-γ and inhibition of IDO activity as well as genetic ablation of IDO prevents development of depressive-like behavior in response to BCG [53]. IDO-dependent degradation of tryptophan leads to production of its metabolite kynurenine, which is further metabolised to quinolinic acid and kynurenic acid, an NMDA-receptor agonist and NMDA-receptor antagonist respectively. Glutamergic dysfunction has been implicated in the development of MDD. In addition, trypophan/kynurenine and kynurenic acid/kynurenine ratios appear to be distorted in individuals with cytokine-induced MDD as well as in otherwise healthy individuals with MDD [48].

We observed pre-existing higher levels of T-cell cytokine production in individuals who subsequently developed depressive symptomatology after exposure to a period of severe stress, i.e. military deployment, suggesting increased T-cell functioning prior to the development of depressive symptoms. In apparent contrast to our data, decreased mitogen-induced T-cell proliferation in vitro and decreased virus-specific T-cell responses in vivo have been observed in individuals who already had developed MDD [54], [55], indicating decreased T-cell function within these individuals. Based on these previous studies, we speculate that depressive symptomatology may decrease T-cell function over time. In this respect it is of interest that IDO can inhibit T-cell function [52]. It is possible that the observed higher capacity of T-cells to produce cytokines in individuals vulnerable to development of depressive symptoms eventually results in upregulation of IDO by peripheral antigen presenting cells. Therefore, one could suggest that in the long run, upregulation of IDO may contribute to the downregulation of T-cell function in depressed individuals.

Exploratory structural equation _odelling, in which exploratory factor analysis is performed within a structural equation _odelling setting [21], was used for reduction of the cytokine data. This advanced statistical method permitted us to reduce the complex dataset into factor scores and to investigate the longitudinal stability of our obtained factor solution. The raw data of T-cell-produced cytokines and monocyte-induced cytokines support our factor solution in which pro- and anti-inflammatory cytokines load on the same factors: we did not observe differences in the direction of the observed change over time between pro- and anti- inflammatory cytokines.

The longitudinal design of our study also allowed us to investigate the effect of military deployment on mitogen-induced cytokine production within the whole sample. Military deployment induced changes in mitogen-induced T-cell cytokine production, T-cell-induced chemokine/IL6 production and innate cytokine production. Interestingly, the direction of the observed change in cytokine production upon stimulation differed between monocytes and T-cells. Innate cytokine production decreased over time. In contrast, the total number of monocytes increased over time for both groups, indicating that the total capacity of a single monocyte to produce cytokines upon stimulation decreased even more strongly over time. The production of T-cell-induced chemokines and IL-6 after stimulation also decreased over time, as well as the number of cytotoxic/suppressor-effector T-cells (CD8+). In contrast, the T-cell cytokine production in response to a mitogen was increased after return from deployment. The observed changes in cytokine production over time were not caused by differences in season during the subsequent assessments influenced our results.

Our group previously reported that mitogen-induced cytokine production was not a stable trait, but differed over time in healthy adolescents, depending on the season of assessment [35]. Nevertheless, after correcting our latent growth models for the seasons in which the subsequent assessments took place, we still observed significant changes in cytokine production over time (data not shown). In addition, the changes in cytokine production over time were not caused by differences in the duration of sample storage in the freezer, since the participants were included in cohorts and therefore the samples from the first assessments have not all been stored longer than samples from the second and third assessments. In addition, if the changes had resulted from degradation of samples then we would have expected that the changes over time were all in the same direction.

It was previously reported that severely stressed individuals [56], including deployed military personnel [57], [58], are at increased risk for later development of medical conditions. It may well be that the observed decreased innate cytokine production after deployment is involved in this increased risk for development of medical conditions, such as bacterial infections. In addition, if the increased capacity of T-cells to produce cytokines lasts for a long period or becomes a stable feature, it may contribute to development of inflammatory conditions, such as atherosclerosis [59], [60].

The observed change in cytokine and chemokine production was long-lasting: 6 months after return the effect was still present. Future studies should investigate how long after return from deployment the observed changes in cytokine production last. This is important since we observed that high T-cell cytokine production is a risk factor for development of depressive symptoms. If a new stressor would occur when the observed T-cell cytokine production has not yet decreased, it may well be possible that the increased T-cell cytokine production facilitates development of depressive symptomatology in response to this new stressor. Indeed, there is evidence that soldiers who have been deployed previously are at increased risk for development of combat-related MDD [61]. However, not all studies have found an association between previous deployment and development of depression in response to a new deployment [62]. One possible confounding factor could be that the interval between deployments differs between studies and between participants within the studies. In future studies, it would be interesting to take the interval between the deployments into account while investigating this relationship.

In addition to the observed pre-existing difference in T-cell cytokine production, individuals with depressive symptoms after deployment also responded differently to the deployment: the group with depressive symptoms after deployment showed a smaller increase in mitogen-induced T-cell cytokine production in response to deployment than the non-depressed group. This observed group difference in the trajectory of T-cell cytokine production over time could not be attributed to a difference in the number of experienced deployment stressors. However, we cannot exclude that potential differences in the experienced subjective severity of these stressors did affect the trajectory of T-cell cytokine production.

The development of depressive symptoms was not associated with the change in T-cell-induced chemokine/IL-6 production and innate cytokine production over time. In addition, innate cytokine production and chemokine production before deployment were also unrelated to development of depressive symptoms

In individuals who already had developed MDD or depressive symptoms, increases in innate cytokine production have repeatedly been reported [2]–[6]. Based on our findings we hypothesize that an increase in mitogen-induced production of innate cytokines may develop only as a consequence of the presence of depressive symptomatology and does not represent a vulnerability factor for development of depressive symptoms.

A limitation of the current study is that depressive symptoms were assessed with self-report questionnaires, and that official MDD diagnoses could therefore not be made. However, the validity of the used SCL-90 depression subscale has repeatedly been investigated and the questionnaire has been found to be a valid and reliable screening tool for the presence of MDD [25]–[27]. Another limitation is that we did not include a non-deployed control group. We interpret the observed changes in mitogen-induced cytokine production over time as consequences of the severe stress experienced during the deployment, but we cannot exclude that other factors contribute to the observed changes in mitogen-induced cytokine production.

The current study is the first to show that the capacity of monocytes and T-cells to produce cytokines and chemokines was altered after exposure to severe stress, i.e. deployment to a combat-zone, until at least 6 months after return. In addition, we found that pre-existing high mitogen-induced T-cell cytokine production is a predictor for the development of depressive symptoms in response to military deployment. We propose that high mitogen-induced T-cell cytokine production may be a vulnerability factor for increased risk for development of depressive symptomatology after exposure to a period of severe stress, such as experienced during military deployment.

## Supporting Information

Table S1 Fit and measurement parameters of the subsequent exploratory structural equation modeling (ESEM)-models.
(DOC)
