# Supplementary material for: Cytokine Production by Leukocytes of Military Personnel with Depressive Symptoms after Deployment to a Combat-Zone: A Prospective, Longitudinal Study
Source: PLoS One. 2011 Dec 14;6(12):e29142. doi: 10.1371/journal.pone.0029142 (PMC3237604; doi:10.1371/journal.pone.0029142)
Supplement: Table S1 — Fit and measurement parameters of the subsequent exploratory structural equation modeling (ESEM)-models. (DOC) [file pone.0029142.s001.doc]

**Online supporting information**

**Table S1. Fit and measurement parameters of the subsequent exploratory structural equation modeling (ESEM)-models.**

|  | Model Fit | | | | | | | | Model Comparison | |
| --- | --- | --- | --- | --- | --- | --- | --- | --- | --- | --- |
|  | *χ^2^* | df |  | CFI | TLI | RMSEA | 90% C.I. RMSEA | SRMR | AIC | BIC |
| 1-factor ESEM model | 5281.581 | 90 |  | .427 | .332 | .239 | .233-.244 | .187 | 38895 | 39117 |
| 2-factor ESEM model | 2267.782 | 53 |  | .700 | .559 | .203 | .196-.210 | .090 | 20241 | 20492 |
| 3-factor ESEM model | 1744.690 | 63 |  | .814 | .691 | .162 | .156-.169 | .050 | 35412 | 35767 |
| 4-factor ESEM model | 1037.845 | 51 |  | .891 | .776 | .138 | .131-.146 | .040 | 34729 | 35143 |
| 5-factor ESEM model | 577.735 | 40 |  | .941 | .844 | .115 | .107-.124 | .027 | 34291 | 34759 |
| 5-factor ESEM model without CD2/CD28-induced IL-4 | 273.136 | 31 |  | .968 | .906 | .088 | .078-.097 | .024 | 34271 | 34704 |
| 4-factor ESEM model without CD2/CD28-induced IL-4 and LPS-induced IL-6 | 270.840 | 32 |  | .962 | .908 | .086 | .077-.095 | .026 | 27052 | 27406 |

Footnote: *χ^2^: Pearson’s Chi Square;* df: degrees of freedom; CFI: comparative fit index; TLI: Tucker-Lewis Index; RMSEA: root mean square error of approximation; 90% CI: 90% confidence interval; SRMR: standardized root mean square residual; AIC: Aikake information criterion; BIC: Bayesian information criterion.
